# Influence of substrate orientation on exciton fine structure splitting of InAs/InP nanowire quantum dots

**DOI:** 10.1186/1556-276X-7-265

**Published:** 2012-05-22

**Authors:** Michał Zieliński

**Affiliations:** 1 Instytut Fizyki, UMK, Grudzia̧dzka 5, Toruń, 87-100, Poland

**Keywords:** Nanowire quantum dots, Excitons, Fine structure splitting, Substrate orientation

## Abstract

In this paper, we use an atomistic approach to investigate strain distributions, single particle and many body electronic properties of InAs/InP nanowire quantum dots with substrate orientation varying from [111] to high-index [119], and compared with [001] case. We show that single particle gap for high-index [11*k*] substrates is increased with respect to [111] and [001] cases, and oscillates with the substrate index due to faceting effects. Surprisingly, the overall shell-like structure of single particle states is preserved even for highly facetted, high-index substrates. On the contrary, we demonstrate that besides two limiting high-symmetry cases, [001] and [111], the bright exciton splitting varies strongly with substrate orientation. For [112]-oriented substrate, the fine structure splitting reaches maximum due to crystal lattice anisotropy despite fully cylindrical isotropic shape of nanowire quantum dot.

## Background

Nanowire quantum dots [1,2] grown by vapor-liquid-solid (VLS) lithography [3,4] have gained a considerable attention over the last few years. VLS growth is a bottom-up process that offers a possibility of tailoring quantum dot diameter, height and the composition by tuning growth conditions. For example, by selecting certain size of gold seed (catalyst) particle, one can control quantum dot diameter with nanometer precision [5] and obtain well-defined, cylindrical shape of the quantum dot. With the use of patterned substrates [5], the location of the catalyst particle itself can be precisely controlled, allowing for the quantum dot positioning and offering, thus, a substantial advantage over traditional Stranski-Krastanov growth mode of self-assembled quantum dots. Further cladding process [6] reduces the surface recombination resulting in good optical properties of nanowire quantum dots; thus, such structures have been analyzed for their potential applications as efficient single photon source [7].

Nanowire quantum dots are typically grown on [111] substrates [4], while the crystal phase can vary between zinc-blende and wurtzite [8,9]. It has been recently shown that, regardless of the crystal phase, the high symmetry of nanowire quantum dots leads to significant reduction of their excitonic fine-structure splitting [10,11], opening possible route for quantum dot-based entangled photon sources. InAs/InP quantum dots emitting at telecommunication relevant wavelengths [12] are particularly interesting for such applications.

In this paper, we use an atomistic approach [13-15] to calculate electronic and optical properties of nanowire InAs/InP quantum dots. Apart from typical [111] substrate growth, we performed our calculations for quantum dots grown on high-index substrates [16] varying from [112] to [119] and compared obtained results with that of [001] substrate case. For this family of ten quantum dots, we have calculated strain distributions, single particle energies, Coulomb integrals, biexciton and trion binding energies [17] and the excitonic (bright and dark splittings) fine structure [18].

## Methods

We performed our calculations for InAs/InP disk-like quantum dot of 2.4-nm height and 18-nm diameter corresponding to typical [5] quantum dot dimensions obtained in VLS lithography. The InAs quantum dot is embedded into the center of InP nanowire of 80-nm diameter and 120-nm height. The nanowire and quantum dots are placed on differently oriented substrates: [001], [111] and high-index substrates [11*k*, with *k*=2,3,…9. The size of the computational domain for strain calculation reaches 32×1^06^atoms resulting in a significant numerical challenge even on modern parallel computers. Such InP buffer thickness and height guarantee convergence of strain distribution and single particle energies well below 1 meV [19]. For calculation of strained atomic positions, we use Keating’s valence force field (VFF) model [13,20]. There are two empirical force constants (*α* and *β*) used in the VFF approach that are fit to reproduce bulk elastic properties (_*C*11__*C*12_ and *C*_44_ bulk elastic constants). At least two fitting schemes and, thus, two VFF parameterizations schemes are possible. In a more traditional [20,21] approach, two *αβ*parameters are obtained directly from bulk elastic *C*_11_ and *C*_12_ constants only, while *C*_44_ constant is not a fitting target but rather comes as an output of the ‘fitting’ process, sometimes resulting in an substantial error of *C*_44_[22]. Such an approach can be, however, well justified for [001] growth where strain properties are dominated by hydrostatic and biaxial strains connected to *C*_11_ and *C*_12_. These constants should be well reproduced, while shear (off-diagonal) strains related to (sometimes poorly described) *C*_44_ are negligible. The latter is unfortunately not true for [111] growth. To overcome this difficulty, recently, a scheme which fits VFF parameters to all tree bulk constants on equal footing has been proposed [23]. We compared and used both of these approaches and obtained identical trends with respect to the substrate index. Hence, in this work, we use former well- established method and present results for one of the parameterizations only. We leave more quantitative study of the differences between two methods for a future work [24].

Once strained atomic positions are obtained, we use them to calculate single particle energies with an empirical tight-binding model under *sp*_3_*d*^5^s^*^ parametrization [25] that accounts for both *d* orbitals and spin-orbit interaction. This model incorporates on-site matrix element correction in a form suitable for non-bulk nanosystem calculation [24] and accounts for atomistic effects such as material interfaces, faceting and crystal lattice symmetry. Finally, once single-particle energy states are found, we calculate electron-electron, electron-hole and hole-hole Coulomb integrals, and the next step is the calculation of many body states using configuration interaction approach [15].

## Results and discussion

### Strain distribution

**Figure 1:**
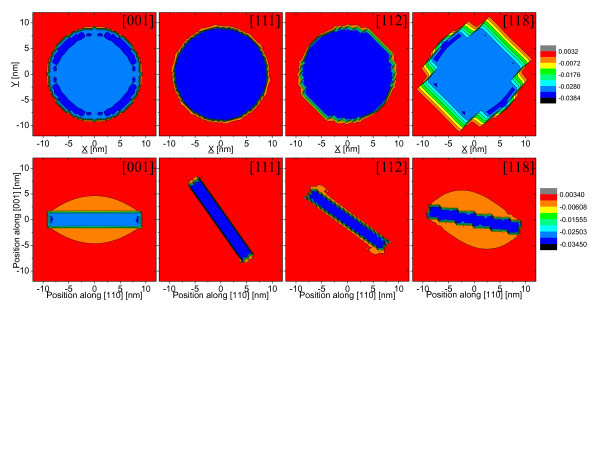
**Hydrostatic strain distribution.** The trace of the strain tensor distribution for InAs/InP nanowire quantum dot (*h* = 2.4 nm, *d* = 18 nm) as a function of substrate orientation. Upper row shows lateral (in-plane) projection through quantum dot center, while lower row projection was calculated for perpendicular [110] plane.

Upper row of Figure 1 shows the hydrostatic strain (the trace of strain tensor) distribution calculated for quantum dots located on differently oriented substrates. The strain is calculated on a plane crossing the quantum dot center and parallel to the quantum dot substrate. Hydrostatic strain distribution is very similar for all studied dots with maximum strain in dot center reaching ≈3.5%, which is consistent with InAs/InP bulk lattice constant mismatch. Despite the presence of low symmetry (zinc-blende) crystal lattice, the overall hydrostatic strain distribution reproduces the disk-like symmetry of the quantum dot shape.

For higher index substrate ([112]), faceting effects become visible on the edges of quantum dots and are very well pronounced for high-[118] index substrate. The characteristic steps in quantum dot strain distribution due to faceting effects are also well visible in Figure 1 (lower row) where hydrostatic strain is plotted on a [110] plane going through dot centers.

**Figure 2:**
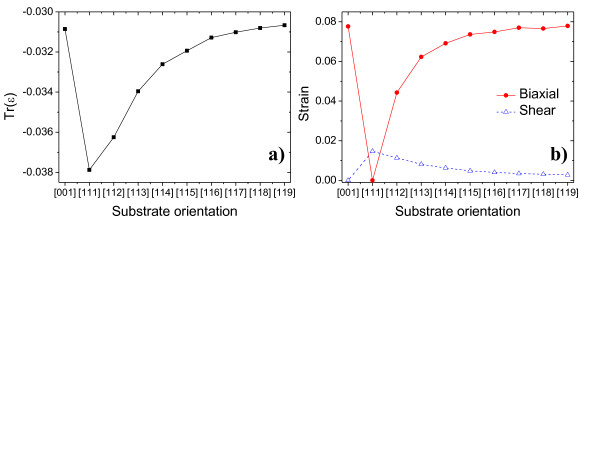
**Different functions of strain at the geometric dot center.** (**a**) The hydrostatic strain *Tr*(*ε*)=_*ε* *xx*_ + _*ε* *yy*_ + _*ε* *zz*_, (**b**) the biaxial $B(\varepsilon )=\sqrt{\left({(\varepsilon_{x}-\varepsilon_{y})}^{2}+{\left(\varepsilon_{y}-\varepsilon_{z}\right)}^{2}+{\left(\varepsilon_{z}-\varepsilon_{y}\right)}^{2}\right)}$ and the average shear strain $S(\varepsilon )=\left(\varepsilon_{\mathrm{xy}}+\varepsilon_{\mathrm{yz}}+\varepsilon_{\mathrm{zy}}\right)/3$ of InAs/InP nanowire quantum dot (*h* = 2.4 nm, *d* = 18 nm) as a function of substrate orientation.

Figure 2 shows different functions of strain calculated at the geometric dot center. The absolute of (a) the hydrostatic strain *Tr*(*ε*)=_*ε* *xx*_ + _*ε* *yy*_ + _*ε* *zz*_for [111] substrate (3.8%) is noticeably larger than [001] substrate (3.1%). We speculate that one could expect even larger difference between [001] and [111] substrates for highly strained InAs/GaAs quantum dots. With increasing substrate index, the magnitude of the hydrostatic strain goes down reaching, for [119] case, the value comparable to that of non-tilted [001] system. Similar trend is observed for (b) biaxial strain defined as $B(\varepsilon )=\sqrt{\left({(\varepsilon_{x}-\varepsilon_{y})}^{2}+{\left(\varepsilon_{y}-\varepsilon_{z}\right)}^{2}+{\left(\varepsilon_{z}-\varepsilon_{y}\right)}^{2}\right)}$, where *x,y,* and *z* correspond to directions defined by crystal axes [100], [010] and [001], respectively. Biaxial strain defined as *B*(*ε*) is largest for flat and disk-like quantum dot located on [001] substrate, while it is exactly zero (by definition and the quantum dot symmetry) for [111] case. On the contrary, function of shear (off-diagonal) strain defined as $S(\varepsilon )=\left(\varepsilon_{\mathrm{xy}}+\varepsilon_{\mathrm{yz}}+\varepsilon_{\mathrm{zy}}\right)/3$ is exactly zero (at the dot center) for [001] substrate quantum dot and reaches maximum for [111], demonstrating the significant role of the [111] biaxial strain for systems grown on [111] substrate.

**Figure 3:**
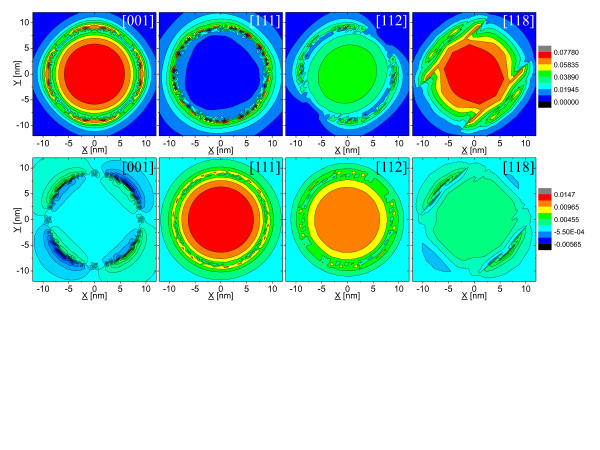
**Lateral (in-plane) projection through quantum dot center for two functions of strain.** The biaxial strain $B(\varepsilon )=\sqrt{\left({(\varepsilon_{x}-\varepsilon_{y})}^{2}+{\left(\varepsilon_{y}-\varepsilon_{z}\right)}^{2}+{\left(\varepsilon_{z}-\varepsilon_{y}\right)}^{2}\right)}$ (upper row) and the average shear strain $S(\varepsilon )=\left(\varepsilon_{\mathrm{xy}}+\varepsilon_{\mathrm{yz}}+\varepsilon_{\mathrm{zy}}\right)/3$ (lower row) of the InAs/InP nanowire quantum dot (*h* = 2.4 nm, *d* = 18 nm) as functions of substrate orientation.

The spatial distribution of *B*(*ε*) or *S*(*ε*) over entire quantum dot spatial domain is more important than one particular value at the quantum dot center. As shown in Figure 3 (upper row) for [001] and [111] substrate orientations, the spatial distribution of *B*(*ε*) is nearly cylindrical, determined by the shape symmetry of the quantum dot, reaching maximum at the dot center for [001] case and highly varying at the quantum dot-matrix interface. On the contrary, it is only the quantum dot-surrounding matrix interface where *B*(*ε*) reaches non-negligible values for [111] case.

There is a strong quantitative difference of biaxial strain distribution between highly symmetric [001],[111] dots and that grown on [11*k*] (*k*=2,3,…9) substrate, where combination of the quantum dot and lattice symmetry results in strong anisotropy of the strain distribution as visible in Figure 3. For such low symmetry system, we may expect large fine structure splitting (FSS) for both bright and dark excitons despite nominally cylindrical symmetry of quantum dot shape.

It must be pointed here that for quantum dots grown on [001] substrate there are also two non-equivalent axes: [110] and [110], with anisotropy clearly visible on shear strain distribution in Figure 3(lower row), suggesting low *C*^2*v*^ symmetry of this quantum dot system. However, due to the lack of wetting layer and disk shape of nanowire quantum dot, there is additional symmetry operation (*S*^4^ rotoinversion [10]), and overall (lattice + dot) symmetry is actually higher than *C*^2*v*^, i.e., *D*^2*d*^. By group theoretical arguments [10,11], we expect [001] substrate quantum dot to have zero bright exciton structure splitting but non-zero dark exciton splitting.

By similar symmetry arguments, one can deduce that [111] substrate quantum dot has *C*^3*v*^ symmetry, and indeed, such symmetry (triangular-like) is well pronounced on biaxial strain plot in Figure 3. System with *C*^3*v*^ symmetry will have zero fine structure splitting both for bright and dark exciton states. We will verify this general analysis by more strict numerical, tight-binding calculation in the next section.

### Single particle states

**Figure 4:**
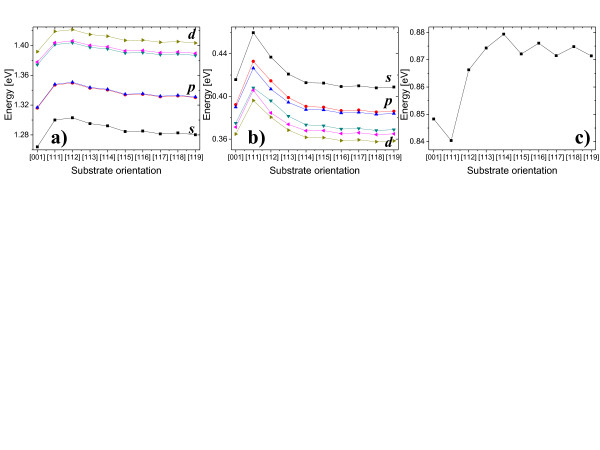
**Single particle energies as functions of substrate orientation.** Several lowest (ground state, black/boxes; first excited state, red/circles; second excited state, blue/triangles, etc.) single particle electron (**a**) and hole (**b**) energies and single particle gap (**c**) of InAs/InP nanowire quantum dot (*h* = 2.4 nm, *d* = 18 nm).

Once strained atomic positions are obtained, we use them to calculate single particle energies with an empirical tight-binding model [24] accounting for *d*-orbitals and spin-orbit interaction. Figure 4a,b shows several lowest electron and hole states as functions of substrate lattice orientation. Surprisingly, despite strong faceting effects visible in strain distribution (Figure 1), the spectra of confined electron states reveal robust shell structure with *p*-shell splitting on the order of 1 meV and s-p level spacing varying between 47 and 52 meV. p-d level spacing is systematically larger (by 6 meV) than that of s-p levels, while the third of *d* states is split from two other by ≈14 meV, a hallmark of disk-like [26], not of lens-type (harmonic oscillator-like) confinement.

The spectrum of confined hole states is more susceptible to a choice of substrate than that of electron states, yet well-visible shell structure is still present as seen in Figure 4b. The splitting of hole *p* shell varies from ≈7 meV for [111] substrate and goes down to 2 meV for high-index and [001] substrates. s-p hole level spacing is in the order of 20 meV, while p-d level spacing is about 15 meV, both smaller than that of electrons due to larger hole effective mass. Splittings within *p* and *d* shells reach maximum for the most anisotropic quantum dot grown on [112] substrate and are generally systematically larger than for electrons, as holes are more affected by anisotropy of confining potential through biaxial strain terms in TB Hamiltonian [25]. Energies corresponding to electron levels, being formed predominately from atomic *s* levels, follow similar trends as hydrostatic strain in Figure 2a that can be understood in terms of simple Bir-Pikus model [22] and single *a*^*c*^ deformation potential. Holes, on the other hand, are build mostly from atomic *p* orbitals and are susceptible to both hydrostatic and biaxial [001] and [111] strains that enter Hamiltonian *via* different deformation potentials (*a*^*v*^*b* and *d*).

Figure 4 shows that, when going from [001] to [111] substrate, the ground hole state is energetically shifted up by 44 meV, while electron ground state is shifted by a smaller amount of about 37 meV only. This difference results in overall reduction of the single particle gap by 7 meV (Figure 4c). Our recent *abinitio* calculations of band deformation potentials [27] suggest gap reduction for [111]-grown quantum dots compared with [001] case, yet the effect should be more pronounced for highly strained InAs/GaAs quantum dot.

Increasing substrate index from [111] to [119] reduces the single particle gap as seen in Figure 4, but even for [119] case, the gap is far from being converged and is larger by about 25 meV compared to [001] system. Interestingly, the gap value reveals oscillations due to faceting effects that were not well pronounced in the electron and hole spectra separately.

In this work, we neglect effects of piezoelectricity. Such approach is well justified [28] for InAs/InP systems due to small strain magnitude as compared to InAs/GaAs systems and partial cancelation of first order piezoelectric terms by second order contributions [29]. More importantly, contrary to straightforward k.p approach [26], piezoelectricity would not alter symmetry of the Hamiltonian which is already well defined by atomistic strained positions entering the TB calculation.

### Coulomb integrals and binding energies

**Figure 5:**
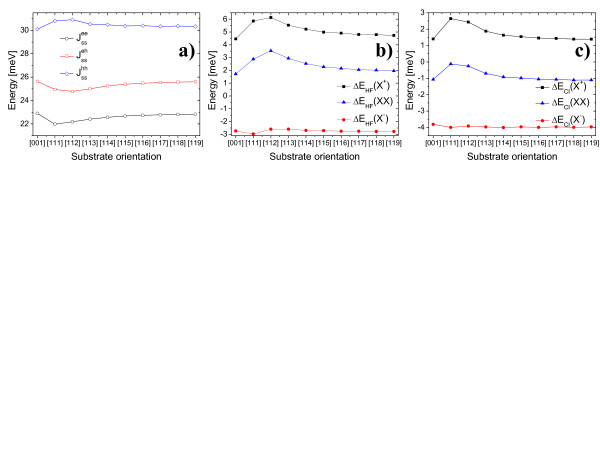
**Coulomb intervals and binding energies.** (**a**) Electron-electron $J_{\mathrm{ss}}^{\mathrm{ee}}$, electron-hole $J_{\mathrm{ss}}^{\mathrm{eh}}$ and hole-hole $J_{\mathrm{ss}}^{\mathrm{hh}}$ Coulomb integrals for electron and hole occupying their ground *s*states of InAs/InP nanowire quantum dot (*h*=2.4 nm, *d*=18 nm) as functions of substrate orientation. (**b**) Biexciton (*XX*) and trion (^*X*−^,^*X* + ^) binding energies calculated by perturbative approach (Hartree-Fock approximation). (**c**) Biexciton (*XX*) and trion (^*X*−^, *X*^ + ^) binding energies calculated by configuration interaction approach (including Coulomb scattering terms up to the *d* shell). _EHF_, binding energy by Hartree-Fock approximation; _ECI_, binding energy by configuration interaction approach.

Figure 5a shows electron-electron $J_{\mathrm{ss}}^{\mathrm{ee}}$, electron-hole $J_{\mathrm{ss}}^{\mathrm{eh}}$ and hole-hole $J_{\mathrm{ss}}^{\mathrm{hh}}$ Coulomb integrals calculated for electron and hole occupying their ground *s* states [17]. There are no faceting effects visible in Figure ??a but rather a smooth change with the increasing substrate index: a manifestation of long-range character of direct Coulomb interaction. Electron-electron and hole-hole interactions are decreased and increased, respectively, for [111] system compared with [001] case; this is consistent with the decreased confinement of electron and increased confinement of hole states (shown above for single particle spectra in Figure 4). One can use *J*^*ss*^ integrals to estimated biexciton (*XX*) and trion (*X*^-^*X*^+^) binding energies at the Hartree-Fock (HF) level [17]:

(1) $$\begin{array}{lll} \Delta E_{\mathrm{HF}}(\mathrm{XX}) & =J_{\mathrm{ss}}^{\mathrm{ee}}+J_{\mathrm{ss}}^{\mathrm{hh}}-2J_{\mathrm{ss}}^{\mathrm{eh}}\; & \;\\ \Delta E_{\mathrm{HF}}\left(X^{-}\right) & =J_{\mathrm{ss}}^{\mathrm{ee}}-2J_{\mathrm{ss}}^{\mathrm{eh}}\; & \;\\ \Delta E_{\mathrm{HF}}\left(X^{+}\right) & =J_{\mathrm{ss}}^{\mathrm{ee}}-2J_{\mathrm{ss}}^{\mathrm{eh}}\; & \; \end{array}$$

Figure 5b shows *XXX*^-^ and *X*^+^ binding energies calculated according to above formulas, suggesting that both *X*^+^ and *XX* are unbound (have positive binding energy). In HF picture, the *X*^+^ binding energy can be roughly estimated to vary between 4 and 6 meV; *XX* binding energies are also positive and reached up to 4 meV, while only *X*^-^ is bound with bounding energy ≈−3 meV. This characteristic ordering of emission lines with increasing energy: *X*^-^*XXX* and *X*^+^ is analogous to that reported in the study of Gong et al. [17] for InAs/InP lens-shaped quantum dot.

However, when correlation effects due to the configuration interaction (mixing) with higher shells (*pd*) are included [15,30,31], the binding energies are shifted towards lower energies as shown in Figure 5c. In particular, *X*^-^ binding energy now reaches ≈−4 meV, while *X*^+^ binding energy is significantly reduced by a semi-rigid shift (≈3.5 meV) for all considered substrate indices. Most importantly, similar correlation correction (≈3.5 meV) results in binding of *XX* complex with binding energy of ≈−1.1 meV for [001] substrate and, interestingly, very small (-0.12 meV) binding energy for quantum dot grown on [111] substrate. With correlation effects accounted for emission lines show following order: *X*^-^*XXX* and *X*^+^. It must be pointed here that detailed ordering of these levels may depend on dot diameter and height [16,17] and should be subject to further studies. The hidden correlation parameter (a measure of correlation effect) [17], defined as $\Delta =\Delta E_{\mathrm{HF}}(\mathrm{XX})-\Delta E_{\mathrm{HF}}\left(X^{-}\right)-\Delta E_{\mathrm{HF}}\left(X^{+}\right)$ for nanowire InAs/InP quantum dot studied in this paper, varies from 1.2 to 1.5 meV, a much larger value than 0.7-0.9 meV reported for self-assembled InAs/InP quantum dots, proving the necessity of the full configuration interaction [15,31] approach for the studies of excitonic complexes in nanowire quantum dots. We point here that small *XX* binding energy for [111]-grown system can be advantageous for certain entangled photon pair generation schemes based on tuning biexciton and exciton energies to resonance [32].

### Fine structure splitting

**Figure 6:**
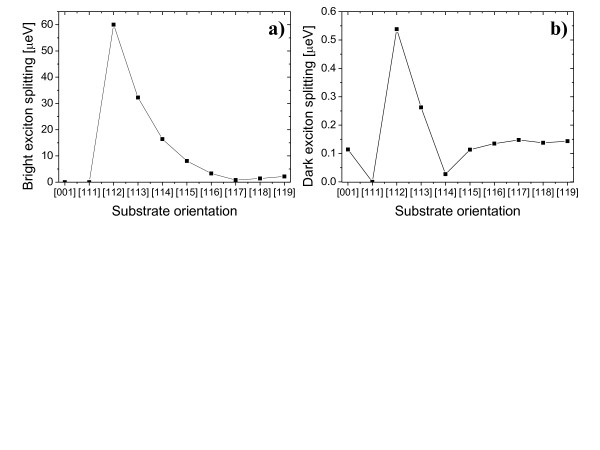
**Exciton fine structure splittings of InAs/InP nanowire quantum dot.** Bright (**a**) and dark (**b**) exciton fine structure splittings of InAs/InP nanowire quantum dot (*h* = 2.4 nm, *d* = 18 nm) as functions of substrate orientation.

Finally, we show the exciton fine structure splitting calculated for nanowire quantum dots as a function of substrate orientation. As shown in Figure 6a, bright exciton structure splitting is exactly zero for quantum dots on [001] and [111] substrates, in agreement with previously mentioned group-theoretical arguments. However, for quantum dot on [112] substrate, there is large bright exciton splitting (60 meV) despite nominally cylindrical shape symmetry of quantum dots. There are no faceting oscillation effects visible on the evolution bright exciton splitting. The large splitting can be attributed to strong in-plane anisotropy of confining potential, as shown earlier on biaxial strain distribution on plot in Figure 2, with $\underline{X}$ and $\underline{Y}$ quantum dot axes corresponding to non-equivalent crystal axes. With increasing substrate index bright exciton FSS is reduced, reaching a minimum of ≈0.7*μ*eV for quantum dot on [117] substrate, then interestingly, it slowly increases reaching up to 4.3 *μ*eV for higher index system ([11*k*] k=20, not shown on the plot). We have checked that alloying effects will reduce bright exciton splitting; however, splitting on the order of 20 *μ*eV still exists for cylindrical _InP0.8_As_0.2_/InP quantum dot grown on [112] substrate. We leave detailed analysis of alloying effects for future research.

Interestingly, splitting of electron and hole *p*-shell cannot be used as simple measure of quantum dot anisotropy and for the straightforward estimation of the fine structure splitting. As mentioned before, electron *p*-shell splitting does not change significantly with substrate index in contrast to bright exciton splitting. The hole *p*-shell changes are more pronounced, and indeed, this splitting reaches a maximum (8 meV) for quantum dot grown on [112] substrate (Figure 3b). Yet, both electron and hole *p*-shell splittings are non-zero for quantum dots on [001] and [111] substrates which have zero bright exciton splitting. Thus a more complicated character the exciton fine structure splitting is revealed, rather than a simple shape anisotropy and the splitting of single particle states.

Last but not least, in Figure 6b, we present dark exciton fine structure splitting as a function of substrate orientation. There is quantitative difference between dark and bright FSS. As predicted by symmetry analysis, dark exciton splitting is exactly zero for *C*^3*v*^ quantum dot on [111] substrate, but non-zero (0.1 *μ*eV) for *D*_2*d*_ quantum dot on [001] substrate. In analogy to bright FSS, the dark FSS reaches maximum (0.5 *μ*eV) for quantum dot on [112] substrate; however, it varies rapidly and stabilizes for high-index substrate at the value (0.15 *μ*eV) similar to [001] case. We note that dark excitons gains non-negligible oscillator strengths for quantum dots on [112]-[115] substrates, but the detailed analysis of dark exciton lifetimes goes beyond the scope of this work.

## Conclusions

We have studied the effects of the substrate orientation on single particle and many body properties of InAs/InP nanowire disk-like quantum dots (h=2.4 nm, d=18 nm). We have shown that, for high-index substrate, there are faceting effects visible in the spatial strain distributions and pronounced in the single particle energy gap. Both electron and hole energies depend on the choice of substrate index, yet the overall shell-like structure is well preserved over wide range of substrate orientations. We calculated the many body properties of nanowire quantum dots, including biexciton and trion binding energy, and concluded that the full configuration interaction treatment is necessary for accurate estimation of excitonic complex binding energies. For disk-like InAs/InP quantum dot on [111] substrate, *XX* binding energy is very small (-0.12 meV) that can be advantageous for the possible generation of entangled photon pairs *via* the recently proposed ‘time reordering’ scheme [32]. Finally, we calculated exciton fine structure splitting and demonstrated that besides two high symmetry cases, [001] and [111], the bright exciton structure splitting varies strongly with substrate orientation. Large bright exciton splitting (60 meV) is predicted for quantum dot grown on [112] substrate despite fully cylindrical geometry of nanowire quantum dot.

We point here that general conclusions for nanowire quantum dot systems should be made after thorough study of many different systems varying with heights and diameters [16] and including alloying effects. We leave this numerically very complex problem for a subject of our future research.

## Competing interests

The author declares that he has no competing interests.

## Author’s information

MZ received a Ph.D. degree in physics from Nicolaus Copernicus University, Torun, Poland in 2006. He was a postdoctoral fellow at the Institute for Microstructural Sciences, National Research Council of Canada, Ottawa for almost 3 years where he performed theoretical research in the area of atomistic, many body calculations of the electronic and optical properties of quantum dots. Since 2009, he has become an assistant professor at the Instytut Fizyki, UMK, Torun, Poland.
